# A LysR Transcriptional Regulator Manipulates Macrophage Autophagy Flux During *Brucella* Infection

**DOI:** 10.3389/fcimb.2022.858173

**Published:** 2022-03-22

**Authors:** Lu Zhang, Siyuan Yu, Xinnuan Ning, Hui Fang, Jie Li, Feijie Zhi, Junmei Li, Dong Zhou, Aihua Wang, Yaping Jin

**Affiliations:** ^1^ College of Veterinary Medicine, Northwest A&F University, Yangling, China; ^2^ Key Laboratory of Animal Biotechnology of the Ministry of Agriculture, Northwest A&F University, Yangling, China

**Keywords:** *Brucella*, LysR-type transcriptional regulators, macrophage, intracellular infection, autophagy

## Abstract

*Brucella*, the intracellular bacteria, have evolved subtle strategies to efficiently survive and replicate in macrophages. However, the virulence effector proteins involved are still unclear. LysR-type transcriptional regulators (lttrs) are the largest regulator family with diverse function in prokaryotes. However, very little is known about the role of LysR regulators in the *Brucella* spp. Here, a *BSS2_II0858* gene, encoded as one of the LysR-type regulators, was studied. We successfully constructed a *BSS2_II0858* deletion mutant, Δ0858, and complementation strain CΔ0858 in *Brucella suis* S2. The cell apoptosis induced by *B. suis* S2 and its derivatives were detected by flow cytometry. The autophagy was then assessed by immunoblot analysis using the IL3I/II and p62 makers. In addition, the autophagy flux was evaluated by double fluorescent labeling method for autophagy marker protein LC3. Our studies demonstrated that *B. suis* S2 and its derivatives inhibited the programmed cell death in early stage and promoted apoptosis in the later stage during infection in RAW264.7 cells. The *BSS2_II0858* gene was found to play no role during apoptosis according to these results. Compared with the wild-type strain, Δ0858 mutant can stimulate the conversion of LC3-I to LC3-II and markedly inhibited the autophagy flux at early stage leading to obvious autophagosome accumulation. This study explored the function of *BSS2_II0858* gene and may provide new insights for understanding the mechanisms involved in the survival of *Brucella* in macrophages.

## Introduction

Brucellosis has been recognized as the most serious common zoonosis globally caused by *Brucella* species (Atluri et al., 2011). In livestock, *Brucella* disrupts reproductive processes causing abortions and infertility and bringing severe economic losses (Boschiroli et al., 2001; Atluri et al., 2011). The central role in the pathogenicity of *Brucella* is its ability to evade the antimicrobial processes and replicate intracellularly in host cells (Boschiroli et al., 2002). After phagocytic uptake and internalization, *Brucella* locates within the *Brucella*-containing vacuole (BCV) to escape killing by the host’s immune system. During transient interaction with the endolysosomal network, the BCV undergo maturation and acidification to trigger the expression of the type IV secretion system (T4SS). The effector of T4SS controls BCV fusion with the endoplasmic reticulum (ER) to establish a replicative BCV (Celli, 2015; Celli, 2019).

Autophagy acts as a housekeeper to maintain cellular homeostasis by eliminating invading bacteria through an autophagic phenomenon: xenophagy (Sharma et al., 2018; Wang and Li, 2020). In eukaryotic cells, intracellular pathogens trigger the xenophagy pathway by forming autophagosomes, fusion with lysosomes, and finally degradation in autolysosomes (Bauckman et al., 2015; Kwon and Song, 2018). This whole dynamic process is referred to as autophagy flux. Microtubule-associated protein light chain 3 (LC3) cleaved by Atg4 to form LC3-I and then conversed into a membrane-bound form (LC3-II) by Atg7/Atg3 in the initiation of autophagy. p62 targets ubiquitinated protein into autophagy vesicles and substrates in autolysosomes *via* interaction with LC3B (Klionsky et al., 2012). Although xenophagy constitutes a powerful host-defense mechanism against invading pathogens, various intracellular microbes have developed dichotomous strategy to subvert xenophagy to establish infection through transcriptional regulators, lipopolysaccharide, and the type IV secretion system (Brumell, 2012; Guo et al., 2012; Wang et al., 2017; Sharma et al., 2018; Celli, 2019).

LysR-type transcriptional regulators (lttrs) comprise the largest family of DNA-binding proteins in prokaryotes. lttrs have an N-terminal DNA-binding helix-turn-helix motif and a C-terminal coinducer-binding domain as conserved structures (Maddocks and Oyston, 2008). These regulators are highly conserved in protein structure and ubiquitous among bacteria, which means that they have a similar diverse function with virulence, motility, metabolism, quorum sensing, scavenging of oxidative stressors, and toxin production (Maddocks and Oyston, 2008). There are more than twenty different LysR regulators in *Brucella*. The gene of BABI_1517, subsequently named *vtlR* was identified as a *Brucella* virulence-associated transcriptional LysR-family regulator. However, very little is known about the role of other LysR regulators in *Brucella* evasion of host autophagy.

In this study, a *lysr* mutant *Brucella* strain (△0858) has been constructed by allelic exchange to investigate the role of *lysr* (*BSS2_II0858*) in *Brucella suis* S2 virulence during infection. The characteristics of autophagy induced by *B. suis* S2, △0858, and C△0858-infected RAW264.7 macrophage cells were systematically described by detecting autophagic flux-related indicators and assessing lysosomal functions.

## Materials and Methods

### Bacterial Strains and Cells

*Escherichia coli* DH5α was purchased from TakaRa and grown in Luria-Bertani (LB) broth at 37°C and 200 rpm. Wild-type *Brucella suis* S2 strain was obtained from the Shaanxi Provincial Institute for Veterinary Drug Control (Xi’an, Shaanxi, China). *B. suis* S2 and its derivatives were grown at 37°C on rich medium tryptic soy agar (TSA) for 72 h or in tryptic soy broth (TSB) with shaking for 48 h. The murine-derived macrophage line RAW 264.7 cell were cultured in DMEM (Hyclone, Logan, UT, USA) supplemented with 10% FBS at 37°C with 5% CO_2_.

### Construction of Lysr Mutant and Complementing Strain

The *BSS2_II0858* gene deletion strains were created by allelic exchange as described previously with minor modifications (Zhi et al., 2021). Briefly, 769-bp upstream fragment, 805-bp downstream fragment, and kanamycin resistance gene were amplified by PCR using primers upstream (lysr)-F-CGGTCCTGACCACCCATTTGCCGTTCTTTT and upstream (lysr)-R-ACTTCAAGAACTCTGTAGCACCGCATTGTTGCGCAAATAACGCTGTCC, K+(lysr)-F-GGACAGCGTTATTTGCGCAACAATGCGGTGCTACAGAGTTCTTGAAGT and K+(lysr)-R-CAACTATGTTAATGCGAGAATGGACAGGTGGCACTTTTCGGGGAAATG, and downstream (lysr3)-F-CATTTCCCCGAAAAGTGCCACCTGTCCATTCTCGCATTAACATAGTTG and downstream (lysr)-R-TGAAGGAAACGACATCGGCGATCAGGCGAT, respectively. The purified upstream, kanamycin resistance, and downstream PCR products were overlapped together using primers upstream (LysR)-F and downstream (LysR)-R, then ligated with the linearized pMD-19T vector to generate pMD19-T-LysR for allelic exchange. The recombined plasmid was electrically transformed into chemically competent *B. suis* S2 cells. Transformants were cultured and selected on TSA with 25 μg/ml kanamycin for 72 h. Colonies were screened and verified by colony PCR amplification using LysR-upF CGGCGCATTGGTATAGGCATTGG and K+(LysR)-R primer pairs and K+(LysR)-F 2-downR GCGTCAGGTGGTTGATGTGC primer pairs to identify the clone mutant *BSS2_II0858* containing inserts of the predicted size for kanamycin.

The *BSS2_II0858* complementation strain was constructed using the expression vector pBBR1-MCS4 by native promoters. Briefly, a region with the lysr promoter and *lysr* gene were amplified by PCR using primers lysr (C)-F-TGCGAAAATTTCCGTTGAAAGGG and lysr (C)-R-TCAAGCGTAGTCTGGGACGTCGTATGGGTATTCCGGTTTGGAATGAACCA. The purified fragment was inserted into pBBR1-MCS4 to form the pBBR1-MCS4-*lysr*. This plasmid was transformed into the Δ0858 strain by electroporation to generate lysr complementation strain (CΔ0858).

### RNA Extraction and Real−Time PCR Analysis

To verify the transcriptional level of the *lysr* gene in mutant and complementation strain, total RNA was extracted from wild-type *B. suis* S2, *BSS2_II0858* mutant, and complementation strains by TRIzol reagent (Invitrogen, Inc., Carlsbad, CA, United States). The bacterial genomic contamination was removed using the TURBO DNA-Free Kit (Ambion Inc, Austin, TX). RT-PCR was performed using GoTaq qPCR Master Mix (Promega Corporation, Wisconsin, USA) to generate products corresponding to *lysr* and 16S rRNA. Primer pairs for *BSS2_II0858* were as follows: LysR real-time F 5′-CGTCATTCAGCATCGCAACC-3′ and LysR realtime R 5′-TGCAACAGGAACGATCACCT-3′. RT-PCR conditions were 95°C for 5 min followed by 30 cycles of 95°C, 60°C, and 72°C for 30 s each. The relative transcription levels were determined by the 2^−ΔΔCt^ method.

### Macrophage Infection

Infection assays of the wild-type strain and its derivatives were performed as described previously (Wang et al., 2016). Briefly, RAW264.7 macrophages were seeded into plates at a density of 5 × 10^5^ cells/ml and cultured for 24 h. The confluent monolayers were inoculated with wild-type *B. suis* S2, *BSS2_II0858* mutant, and complementation strains at a multiplicity of infection (MOI) of 100. Also, cell plates were centrifuged at 500*×g* for 10 min for bacterial sedimentation and culture for 1 h for bacterial infection. Cells were then washed three times to remove extracellular bacteria and further incubated with medium containing 50 µg/ml of gentamicin for 1 h to eliminate extracellular bacteria. After washing for three times, the culture medium was replaced by a medium with 25 µg/ml of gentamicin. At the desired time points, the cells were subjected to the following procedures.

### Flow Cytometry Analysis

The flow cytometry analysis was performed following the standard protocol. Briefly, after 24- or 48-h bacterial infection, different sets of RAW264.7 were washed three times with cold PBS by centrifugation. Cells were treated with PI and Annexin V-FITC for 30 min at 4°C in the dark. The cells were then analyzed by flow cytometry (EPICS Altra, Beckman Coulter Cytomics Altra). The scatter plots of PI fluorescence (*y*-axis) vs. FITC fluorescence (*x*-axis) were primed.

### Western Blotting

Western blotting analysis was performed to verify the complementation strain CΔ0858 and to detect the expression of autophagy-related proteins in *Brucella*-infected cells. For verification, the complementation strain, the fresh cultural *B. suis*. S2, Δ0858, and CΔ0858 strains were harvested and washed in phosphate-buffered saline. After centrifugation at 8,000*×g* for 5 min, the pellets were resuspended in SDS-PAGE loading buffer. For autophagy analysis, the infected cell were collected at specific times, and then lysed with RIPA buffer (Sigma-Aldrich Corp., St. Louis, MO, USA) on ice for 30–45 min. Supernatants were collected by centrifugation at 14,000 rpm for 15 min in 4°C. Protein concentrations were determined by BCA assay and then resuspended in SDS-PAGE loading buffer. All samples were heated at 100°C for 5 min. Proteins were separated by SDS-PAGE and transferred onto PVDF membrane. The blots were blocked for 2 h at room temperature with 5% skim milk, and then probed overnight with mouse anti-HA monoclonal antibody (1:2,000, Sigma), anti-LC3 antibody (1:1,000, Sigma L7543), or β-actin (1:1,000, Beijing CWBIO, Beijing, China) as a loading control at 4°C. After washing, the membranes were incubated with secondary antibody conjugated to horseradish peroxidase at room temperature for 1 h. Immunodetection of proteins was visualized through ECL=enhanced chemiluminescence kit (Millipore Corp, Bedford, MA). The blots were visualized using the Gel Image System (Tannon, Biotech, Shanghai, China) and quantified by ImageJ Software. All bands were normalized to a loading control.

### Immunofluorescence Assay

After 24 or 48 h infection with bacteria, different sets of RAW264.7 were washed twice with cold PBS and then fixed with 4% paraformaldehyde. After incubation with 0.25% Triton X-100, slides were first incubated with goat anti-*Brucella* polyclonal antibody (1:100), rabbit anti-LC3B polyclonal antibody (Abcam, Cambridge, UK 1:300), and then stained with donkey anti-goat Alexa Fluor 555 (1:1,000) or donkey anti-rabbit FITC (1:500 dilution, Invitrogen). The nuclei were stained with 100 µl DAPI. Images were conducted on the Nikon A1R-Si confocal microscope system. Assays were performed in triplicate.

### Autophagy Flux Analysis

Autophagy flux was investigated using the reporter plasmid pCMV-mCherry-GFP-LC3B (Beyotime Biotechnology, China). RAW264.7 cells were transfected with pCMV-mCherry-GFP-LC3B using Lipofectamine 8000 (Beyotime Biotechnology, China) for 24 h. The cells were then infected following macrophage infection procedure, and the fluorescent was observed with a confocal microscope (TCS SPE, Leica, Germany). Representative cells were selected and photographed.

### Statistical Analysis

Data were imported into GraphPad Prism 6.0 software (GraphPad Software Inc., La Jolla, CA, USA) for analysis. Statistical significance was determined using two-way ANOVA followed by Holm–Sidak’s multiple test or Chi-square test. A probability (*p*)-value of <0.05 was considered statistically significant.

## Results

### The Lysr Mutant and the Complementation Strain Were Successfully Constructed

**Figure 1:**
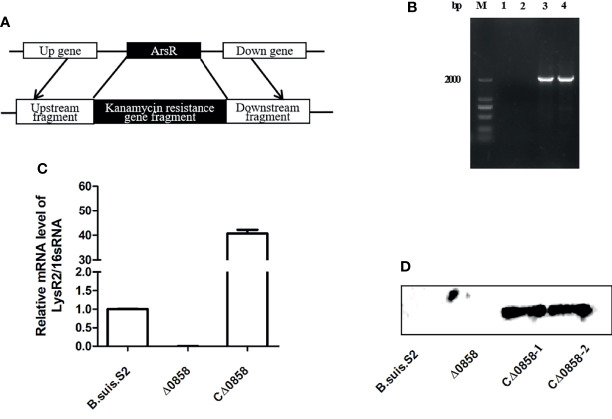
*BSS2_II0858* deletion mutant and complementation strains were successfully constructed. **(A)** Schematic of the construction of the *BSS2_II0858* mutant strain. **(B)** Identification of the *BSS2_II0858* mutant by PCR amplification. Lanes: 1, the gene upstream of *BSS2_II0858* and the kanamycin resistance gene in *B. suis* S2; 2, the gene downstream of *BSS2_II0858* and the kanamycin resistance gene in *B. suis* S2; 3, the gene upstream of *BSS2_II0858* and the kanamycin resistance gene in the *BSS2_II0858* mutant strain; and 4, the gene downstream of *BSS2_II0858* and kanamycin resistance gene in the *BSS2_II0858* mutant strain. **(C)** qRT-PCR confirmed the mRNA expression of *BSS2_II0858* in the *B. suis* S2, *BSS2_II0858* mutant, and complementation strains. **(D)** The inducible expression of flag-tagged *BSS2_II0858* was detected by Western blotting.

To investigate the potential function of *lysr* gene in *Brucella suis* S2, the *BSS2_II0858* deletion and the *BSS2_II0858* complementation strains were constructed by an allelic exchange method. The lysr mutant was verified by PCR amplification of the upstream fragment, downstream fragment, and kanamycin resistance gene fragment using respective primers (**Figure 1A**). The lysr mutant was verified by PCR amplification. The results indicated that a 2,058-bp fragment containing the gene upstream of *BSS2_II0858* and the kanamycin resistance gene and a 2,034-bp fragment containing the gene downstream of *BSS2_II0858* and the kanamycin resistance gene were not amplified from the *B. suis* S2 but were amplified from the *BSS2_II0858* mutant strains (**Figure 1B**). In addition, the complementation strain Δ0858 was verified by RT-PCR and Western blotting. The result showed that *BSS2_II0858* ORF fragment could be detected in wild-type and CΔ0858 strains but not in Δ0858 strain (**Figure 1C**). The HA-tags were successfully expressed in CΔ0858 strains but not in wild-type and Δ0858 strains (**Figure 1D**). These observations indicated that the *BSS2_II0858* mutant and complementation strains were successfully constructed.

### Effect of *B. suis* S2 on RAW 264.7 Viability

**Figure 2:**
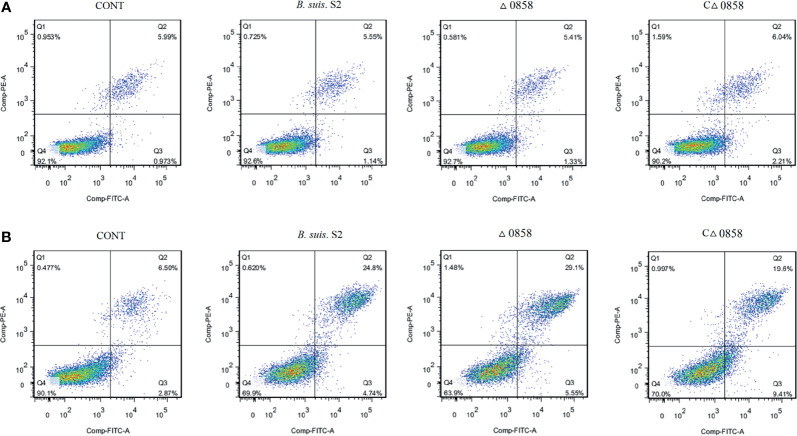
Representative flow cytometry dot plots of apoptosis by Annexin V/PE staining. **(A)** The apoptosis of wild-type *B. suis* S2, *BSS2_II0858* mutant, and complementation strain-infected RAW 264.7 were assayed using Annexin V/PE staining after a 24-h challenge. **(B)** The apoptosis of wild-type *B. suis* S2, *BSS2_II0858* mutant, and complementation strain-infected RAW 264.7 were assayed using Annexin V/PE staining after a 48-h challenge. The results are expressed from 3 independent experiments.

To assess cell apoptosis in WT *B. suis* S2 and its derivatives in infected RAW264.7 cells, apoptosis rate was measured by flow cytometry in combination with Annexin V/PI double straining (**Figure 2A**). The results indicated that WT *B. suis* S2 and its derivatives inhibited macrophage late (PI+/Annexin-V+) but not early (PI−/Annexin-V+) apoptosis after 24 h infection (**Table 1**). However, when the RAW 264.7 cells were infected with *Brucella* for 48 h, the average proportion of progressed apoptotic cells increased significantly compared with mock infected cells. The results also show that no significant apoptosis rate was found among *Brucella*-challenged groups (**Table 1**).

**Table 1:** Flow cytometry assays for cell apoptosis after *B. suis* S2 and derivative infection at 24 and 48 h.

Group	Time after infection (h)	Early apoptotic cells (%)	Progressed apoptotic cells (%)	Survival cells (%)
Noninfection	24	1.07 ± 0.18	6.84 ± 0.74^*^	91.13 ± 1.00
*B. suis.* S2		1.37 ± 0.32	5.51 ± 0.19	92.37 ± 0.40
△lysr		1.52 ± 0.44	5.85 ± 0.65	91.77 ± 1.14
C△lysr		2.11 ± 0.78	6.13 ± 0.15	90.80 ± 0.95
Noninfection	48	2.57 ± 0.64	7.02 ± 1.58^*^	89.87 ± 2.16^*^
*B. suis* S2		6.27 ± 3.25	30.50 ± 4.94	62.07 ± 7.52
△lysr		5.78 ± 0.78	25.60 ± 7.31	67.50 ± 6.67
C△lysr		8.97 ± 1.04	25.23 ± 9.33	64.57 ± 8.73

Data represent the means ± standard deviations from 3 replicates. The asterisk (^*^) represents significant differences (p < 0.05) in cell apoptosis in RAW264.7 cells infected by Brucella compared with that in uninfected cells.

### Mutant Lysr Strain Infection Enhanced Autophagy

**Figure 3:**
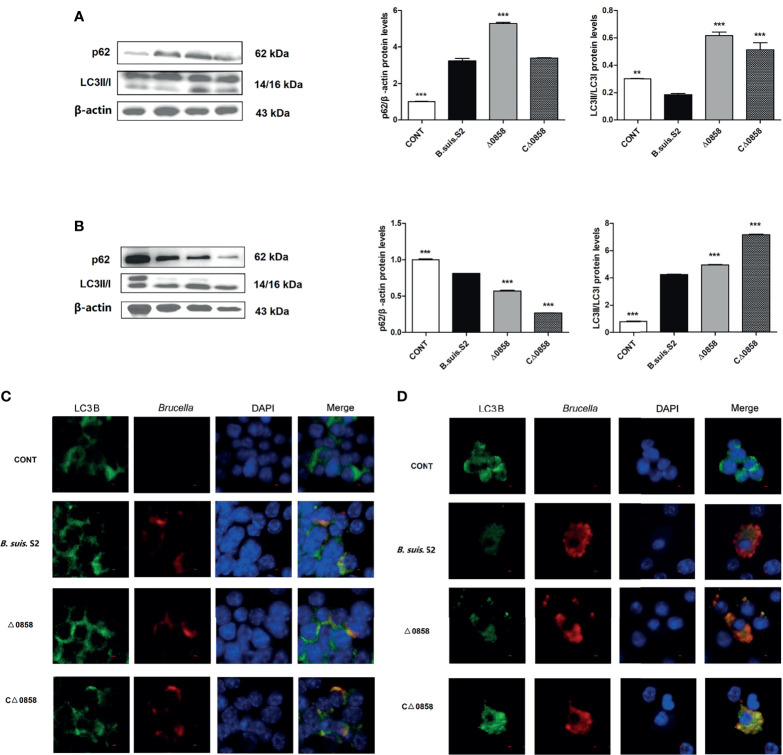
*BSS2_II0858* mutant induced the occurrence of autophagy. **(A)** Light chain 3II (LC3II) and p62 proteins level in WT, *BSS2_II0858* mutant, and complementation strain-infected RAW 264.7 at 24 h. **(B)** LC3II and p62 proteins level in WT, *BSS2_II0858* mutant, and complementation strain-infected RAW 264.7 at 48 h. Data represent the mean ± standard deviations from 3 independent experiments: ^**^ *p* < 0.01, ^***^ *p* < 0.001. **(C, D)** Formation of LC3 puncta (green) and intercellular *Brucella* (red) was observed under the confocal microscope after *Brucella* infection at 24 **(C)** and 48 h **(D)**. Scale bars: 2 µm.

Autophagy caused by WT *B. suis* S2 and its derivatives in RAW264.7 cells was explored. The results revealed that the conversion of ILC3I to LC3II was increased in *BSS2_II0858* mutant strain infected at 24 and 48 h. The ratio of LC3-II/LC3-I was notably higher in the Δ0858 strain- than in the WT *B. suis* S2-infected groups at 24 and 48 h. The ratio of LC3-II/LC3-I in the WT strain-infected group was significantly decreased at 24 h but increased after a 48-h challenge. In contrast, the expression of p62 increased significantly at 24 h but decreased at 48 h among all challenged groups compared with the control group. Interestingly, Δ0858 strain induced higher p62 expression at 24 h but lower at 48 h compared with the WT strain infection (**Figures 3A, B**). As expected, confocal microscopy analysis showed that *Brucella* could induce autophagosome formation in the infected macrophages, and the LC3B puncta formation was further increased in the Δ0858 strain-infected group (**Figures 3C, D**).

### Autophagy Flux Was Blocked by *BSS2_II0858* Mutant Strain

**Figure 4:**
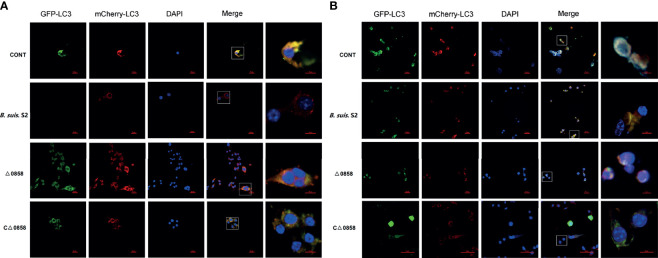
*BSS2_II0858* mutant blocked autophagy flux. **(A)** Representative confocal images of the RAW 264.7 grown on coverslips were transfected with mCherry-GFP-LC3 for 24 h, then infected with WT, *BSS2_II0858* mutant, and complementation strains at 24 **(A)** and 48 h **(B)**.

In order to further evaluate if *BSS2_II0858* mutant strain manipulates autophagy, autophagic flux was assessed using the mCherry-EGFP-LC3B flux assay in RAW26.47 cells. The results indicated that red puncta fluorescence increased after 24 and 48 h in WT *B. suis* S2-inoculated cells. Whereas, yellow puncta fluorescence increased after a 24-h challenge in the Δ0858 strain-infected group (**Figures 4A, B**). This outcome again suggested that Δ0858 strain blocked autophagy flux activated during infection.

### Discussion

Xenophagy, a specialized form of autophagy, could act as an innate immune response to defend against invading pathogens (Wang and Li, 2020). However, intracellular bacterial pathogens *Brucella* have evolved unique mechanisms to counteract host xenophagy machinery for invasion and replication, although the detailed mechanisms are not well known (Hamer et al., 2014; Bauckman et al., 2015). Therefore, exploring the interaction between *Brucella* and autophagy in macrophage is important. lttrs is the largest regulator family in prokaryotes (Zaim and Kierzek, 2003; Bernier et al., 2008; Zhou et al., 2010; Koentjoro et al., 2018). However, very little is known about the role of lysr regulators in the *Brucella* spp. In this study, we mutated a lttrs (*BSS2_II0858*) gene in wild-type *B. suis* S2 and investigated the role of *BSS2_II0858* in autophagy flux.

Gene disruption, gene truncation, gene mutation, complementation, and overexpression are common methods to analyze gene function in prokaryotes (Russo et al., 2009; Zhi et al., 2020). Allelic replacement is a mature method used in the construction of gene mutant strains. In this study, we mutated *BSS2_II0858* gene by allelic replacement and complemented it by using a suitable expression vector. We found that apoptosis rate in macrophages was not affected by the deletion of *BSS2_II0858*. *Brucella* survival and replication inside host cells are critical for the establishment of chronic infection (De Bolle et al., 2015). Recent studies have shown that smooth *Brucella suis* are able to inhibit apoptosis, whereas rough *Brucella* strains undergo apoptotic cell death (Wang et al., 2016). In our study, WT, *BSS2_II0858* mutant, and complementation strains inhibited the macrophage progressed cell death after a 24-h challenge. In contrast, three strains induced dramatically programmed cell death after 48-h infection. Therefore, the inhibition and promotion of apoptosis occurs during the entire process of *Brucella* infection. However, there were no differences among the WT, *BSS2_II0858* mutant, and complementation strains (**Figure 2**).

LC3-II and P62 are the most common used as autophagic markers for autophagosome due to they can locate the autophagosome membrane (Lamark et al., 2017). The amount of LC3I conversion to LC3II indicates the intensity of autophagy (Mizushima and Yoshimori, 2007; Schaaf et al., 2016). In this study, the *BSS2_II0858* mutant strain but not the WT and complementation strains enhanced LC3II expression after 24 and 48-h infection with *Brucella* and its derivatives (**Figure 3**). P62 is known to mediate the degradation of its recognition substrate by autophagy and can be used as a marker of autophagic flux (Lamark et al., 2017). Based on autophagy detection guidelines, an increase in LC3 conversion but not in p62 expression represents the upregulation of autophagic flux, whereas an increase in LC3 conversion and p62 expression represents the inhibition of autophagic flux. The mCherry-GFP-LC3 is an efficient assay to detect the levels of autophagic flux (Geng et al., 2020). Our results show a high expression of p62 and yellow fluorescence accumulation after *BSS2_II0858* mutant strain infection (**Figure 3**). The results indicated that the mutant lysr strain enhances autophagy and blocks the autophagy flux. The ability to regulate gene expression to adapt to the host’s intracellular environment is a crucial component of bacterial virulence. Lysr-type transcriptional regulators comprise the largest family of DNA-binding proteins in many bacteria. LysRs play an important role in the regulation of virulence genes in *Salmonella enterica* serovar Typhimurium (Caldwell and Gulig, 1991), *Agrobacterium tumefaciens* (Doty et al., 1993), *Pseudomonas aeruginosa* (Hamood et al., 1996), and *Vibrio cholerae* (Golberg et al., 1991). Three LysR family regulators LysR12, LysR13, and LysR18 have been identified to be related to *Brucella* 16M virulence (Haine et al., 2005). The expression of abcR2 small RNA, which mediates host–pathogen interactions, is controlled by LysR-type transcriptional regulator VtlR in *Brucella abortus.* VtlR is also required for *B. abortus* to survive and replicate in macrophages (Sheehan et al., 2015). In this study, we explored the function of BSS2_II0858 gene and found that *B. suis* S2 BSS2_II0858 manipulated host autophagy flux. These results may provide new insights for understanding the mechanisms involved in the survival of Brucella in macrophages.

## Data Availability Statement

The original contributions presented in the study are included in the article/supplementary material. Further inquiries can be directed to the corresponding authors.

## Author Contributions

YJ and AW: conceptualization. LZ: project administration, data curation, investigation, and writing. SY and XN: data curation. HF and JiL: software. FZ and JuL: validation. DZ: methodology, writing, reviewing, and editing.

## Funding

This project was supported by the China Postdoctoral Science Foundation (2021M702687), Guangdong Basic and Applied Basic Research Foundation (2021A1515110471), Natural Science Basic Research Plan in Shaanxi Province of China (2020JM-164), National Key R&D Program of China (2018YFD0500900), and the National Natural Science Foundation of China (31672584).

## Conflict of Interest

The authors declare that the research was conducted in the absence of any commercial or financial relationships that could be construed as a potential conflict of interest.

## Publisher’s Note

All claims expressed in this article are solely those of the authors and do not necessarily represent those of their affiliated organizations, or those of the publisher, the editors and the reviewers. Any product that may be evaluated in this article, or claim that may be made by its manufacturer, is not guaranteed or endorsed by the publisher.
